# Comparing terminology mappings to ICD-10 coded data in Discharge Abstract Database (DAD) in Alberta, Canada

**DOI:** 10.23889/ijpds.v9i5.2896

**Published:** 2024-09-18

**Authors:** Namneet Sandhu, Bing Li, Danielle A Southern, Glenda Tower, Debbie Onos, Anita Lochhead, Sarah Whittle, Hude Quan

**Affiliations:** 1 University of Calgary; 2 Alberta Health Services

## Introduction

Coding has become burdensome to healthcare systems due to patient complexity and resource requirements. In Alberta, Intelligent Medical Objects (IMO), an interface terminology mapping product, is integrated within the new province-wide Clinical Information System, named Connect Care (CC), to support documentation by clinicians and map clinical terminologies to ICD-10. This study evaluates comparability of terminologies mapped ICD-10 codes to the ICD-10 codes in DAD.

## Approach

We conducted a retrospective analysis by linking acute care hospital DAD with CC between April 2021 and December 2023. The primary outcome was the level of agreement between ‘hospital problem list’ of CC and DAD for ICD-10-CA codes at a 3-digit level. The number of diagnoses and rate of unspecified codes were also compared. The outcome measures were stratified by physician specialty, hospital type and location, and length-of-stay (LOS). 

## Results

A total of 498,834 unique hospital records were linked. The average level of agreement between CC and DAD at 3-digit level of ICD-10-CA code was 43.4%. The average number of diagnoses captured in CC (3.91) was slightly lower than DAD (4.06), and the average rate of unspecified codes was higher in CC (26.4%) compared to DAD (23.0%). The level of agreement varied by specialty and length-of-stay with specialties with more complex patients and longer lengths-of-stay having the lowest agreement (43% for generalists and internal medicine and 33% for LOS >3 months).

## Conclusion

Level of agreement between CC and DAD for ICD-10 data was identified as low, indicating significant disparities between terminology mappings and the coding process.

